# *H. pylori* May Not Be Associated with Iron Deficiency Anemia in Patients with Normal Gastrointestinal Tract Endoscopy Results

**DOI:** 10.1155/2014/375915

**Published:** 2014-12-31

**Authors:** Tayyibe Saler, Şakir Özgür Keşkek, Sibel Kırk, Süleyman Ahbab, Gülay Ortoğlu

**Affiliations:** ^1^Department of Internal Medicine, Umraniye Training and Research Hospital, 34767 Istanbul, Turkey; ^2^Department of Internal Medicine, Numune Training and Research Hospital, Yüreğir, 01240 Adana, Turkey; ^3^Department of Internal Medicine, Haseki Training and Research Hospital, 34087 Istanbul, Turkey

## Abstract

*Background*. The aim of this study was to investigate the association between iron deficiency anemia and *H. pylori* in patients with normal gastrointestinal tract endoscopy results. *Materials and Methods*. A total of 117 male patients with normal gastrointestinal tract endoscopy results were included in this retrospective study. The study and control groups included 69 and 48 patients with and without iron deficiency anemia, respectively. The prevalence of *H. pylori*, the number of RBCs, and the levels of HGB, HTC, MCV, iron, and ferritin were calculated and compared. *Results*. There was no statistically significant difference found between the groups according to the prevalence of *H. pylori* (65.2% versus 64.6%, *P* = 0.896). Additionally, the levels of RBCs, HGB, HTC, MCV, iron, and ferritin in the patients in the study group were lower than those in the control group (*P* < 0.05). Finally, there was no association between iron deficiency anemia and *H. pylori* (OR 1.02, Cl 95% 0.47–2.22, and *P* = 0.943). *Conclusion*. *H. pylori* is not associated with iron deficiency anemia in male patients with normal gastrointestinal tract endoscopy results.

## 1. Introduction

Anemia is the most common disorder of the blood and is characterized by a decrease in the number of red blood cells or a less-than-normal quantity of hemoglobin in the blood. The hemoglobin value below which anemia is defined varies, although the World Health Organization (WHO) hemoglobin thresholds of less than 13 g/dL for men and less than 12 g/dL for women [1] are the most common definitions used for anemia.

Iron deficiency anemia is the most common form of anemia worldwide. It is a global public health problem affecting both developing and developed countries, with major consequences for human health as well as social and economic development. The causes of iron deficiency anemia include inadequate iron intake, chronic blood loss, and impaired iron absorption. Blood loss from the gastrointestinal tract is the most common cause in men and postmenopausal women [2, 3].

*Helicobacter pylori* is a Gram-negative bacterium that colonizes human gastric mucosa, leading to chronic antral gastritis and peptic ulcer disease. It is also associated with serious diseases, including gastric cancer and gastric mucosa-associated lymphoid tissue lymphoma.* H. pylori* remains one of the most common infections in the world, with an estimated 50% of the world's population being carriers of the bacterium [4, 5].

Previous studies have shown that* H. pylori* colonization of the gastric mucosa may impair iron uptake and increase iron loss, potentially leading to iron deficiency anemia [6, 7]. There have been no studies performed in patients with intact gastric mucosa. Therefore, in this study we aimed to investigate the association between* H. pylori* and iron deficiency anemia in patients with normal gastrointestinal tract endoscopy results.

## 2. Materials and Methods

For this study, a total of 1251 patient files were analysed. There were 906 patients with sufficient data for this study, but patients with chronic diseases, under 18 years old, diagnosed with anemia other than iron deficiency anemia, abnormal gastrointestinal tract endoscopy results, malignancies, parasitosis, positive fecal occult blood tests, and those taking medications that affect* H. pylori*, blood levels, or iron levels, as well as females, were excluded. Finally, 117 male patients with normal gastrointestinal tract endoscopy results were included in this study. The study group consisted of 69 patients with iron deficiency anemia, and the control group consisted of 48 patients without anemia. Females were excluded due to a high prevalence of anemia due to other causes, such as menstruation.

Iron deficiency anemia was defined according to the World Health Organization (WHO) criteria [1], which define anemia as a hemoglobin level of <13 g/dL in men and <12 g/dL in women. The definition of iron deficiency anemia was accepted as being when the serum iron was <37 *μ*g/dL and ferritin was <13 ng/mL. The complete blood counts, serum iron levels, and ferritin concentrations of all of the patients were reported, and the peripheral blood samples were evaluated. Additionally, the complete blood counts were measured using the Sysmex XE 2100i (Japan) by fluorescence flow cytometry. The serum iron levels and ferritin concentrations were measured with the Roche C-601 analyser tract (Japan) using an electrochemiluminescence immunoassay at the institute. A Fujinon EG-590 WR HD (Saitama, Japan) model device was used for the gastrointestinal tract endoscopy procedures (esophagogastroduodenoscopy and colonoscopy) in those years at the institute.

Biopsy samples taken from the antrum during the operation were evaluated after being stained with PAS-AB or modified Giemsa by an experienced pathologist. According to the patient files, we reported that the analyses of* H. pylori* were made via histological examination and the rapid urease test (CLO test: Campylobacter-like organism test). According to the CLO test (Delta West Bentley, WA, Australia), the change in the colour of the test from yellow to red was accepted as a positive result [8]. The prevalence of* H. pylori* infections, the number of RBCs, and the levels of HGB, HTC, MCV, iron, and ferritin were calculated and compared in both groups.

MedCalc 12.7 software program (MedCalc Belgium) was used for statistical analysis. Categorical measurements were reported as number and percentage. Quantitative measurements were reported as the mean ± SD (Standard Deviation). Kolmogorov-Smirnov test was used to show the normal distribution of quantitative measurements. Chi square test was used to compare categorical measures and frequency of metabolic syndrome between the groups. *t*-test or Mann-Whitney *U* tests were used for comparison of quantitative measurements between the two groups. An odds ratio was used to analyse the degree of association between* H. pylori* and iron deficiency anemia. The level of statistical significance was set as 0.05 in all tests.

## 3. Results

The characteristics of the groups are shown in Table 1, and the mean ages are comparable in both groups: 37.4 ± 10.4 and 40.2 ± 10.0 in the study and control groups, respectively (*P* = 0.143). There was no statistically significant difference between the groups according to the prevalence of* H. pylori* (65.2% versus 64.6%, resp.; *P* = 0.896). Not surprisingly, the RBC, HGB, HTC, MCV, iron, and ferritin levels of the patients in the study group were lower than those in the control group. There were no statistically significant differences between the RBC, HGB, HTC, MCV, iron, and ferritin levels in the patients with or without* H. pylori* infections according to the presence or absence of iron deficiency anemia (*P* > 0.05; Tables 2 and 3). When we mixed the patients (anemic and nonanemic) and divided them according to the* H. pylori* infections, we saw that there was no statistically significant difference (*P* > 0.05; Table 4). Finally, there was no association between iron deficiency anemia and* H. pylori* (OR 1.02, Cl 95% 0.47–2.22, *P* = 0.943).

## 4. Discussion

In this study, we have shown that* H. pylori* is not associated with iron deficiency anemia in men with normal gastrointestinal tract endoscopy results. Iron deficiency anemia is a common health problem in the general population [1, 2]. Similarly,* H. pylori* is a common gastrointestinal tract infection that affects a majority of the population. Guidelines on iron deficiency anemia have confirmed the etiological role of* H. pylori*, but the relationship remains controversial. Some previous studies have reported that* H. pylori* is associated with iron deficiency anemia; since* H. pylori* colonization in the gastric mucosa may disturb some functions of the mucosa, it leads to a decrease in iron absorption and increases iron loss [6, 7]. This is an excellent description for the results reported in these studies; however, these results may be acceptable only for patients with abnormal gastrointestinal tract endoscopy.

According to our opinion, iron deficiency anemia and* H. pylori* infections may be a coincidence because both of the diseases are highly prevalent. Moreover, there are many causes that lead to iron deficiency anemia, such as malnutrition, vitamin deficiencies, chronic disorders, infections, and conditions associated with chronic blood loss [9, 10]. Therefore, we planned our study in patients with normal gastrointestinal tract endoscopy results and found no association. Furthermore, we have shown no difference between the RBC, HGB, HTC, MCV, iron, and ferritin levels in the patients with or without* H. pylori* infections in both groups. To our knowledge, this is the first study that investigates the association between iron deficiency anemia and* H. pylori* in patients with normal gastrointestinal tract endoscopy. Male gender in this study may constitute a bias; however, we planned this study with males because women have greater risks for iron deficiency anemia and the majority of them are due to menstruation bleeding. Consequently, we could develop incorrect results if we included women in this study.

According to the literature, there are some studies which have shown the benefits of* H. pylori* treatment on iron deficiency anemia. For example, improved iron deficiency was reported after the eradication of* H. pylori* [11, 12]. In Malik et al.'s study, they have shown that the eradication of* H. pylori* resulted in a significantly better response to oral iron supplementation among* H. pylori* infected pregnant women with iron deficiency anemia [11]. Nevertheless, gastrointestinal endoscopy was not performed in this study; additionally, antiulcer treatment was given to the patients in the study group. In another study, Huang et al. reported that* H. pylori* eradication therapy combined with iron administration is more effective than iron administration alone for the treatment of iron deficiency anemia [12]. They also stated that bismuth based triple therapy has a better response in terms of increased hemoglobin and serum ferritin concentrations than proton pump inhibitor based triple therapy. It is understood that all patients in their study had gastrointestinal problems because they were given bismuth or a proton pump inhibitor based triple therapy. In such cases, iron deficiency anemia is an expected condition due to the impaired mucosa, but in our study all patients had intact mucosa.

Hsiang-Yao et al. studied 882 patients in Taiwan and showed no significant association between chronic* H. pylori* infections and anemia. The sample size of their study was larger, but they did not exclude most of the concomitant conditions. Moreover, gastrointestinal system endoscopy was not performed for all of the patients [13]. Qu et al. performed a meta-analysis of observational studies and randomized controlled trials, and they concluded that iron deficiency anemia could not specifically be related to* H. pylori* infections. Moreover, they did not recommend a strategy of population-based screening and treatment for* H. pylori* infections to prevent iron deficiency anemia [14].

In the present study, iron deficiency in patients with normal gastrointestinal system endoscopy results may be associated with lifestyle, for example, inadequate or improper nutrition and excessive drinking of tea and/or coffee.

The small sample size is a limitation in this study; however, a total of 1251 patient files were analysed at the baseline. We excluded most of the patients due to the wide exclusion criteria, although the wide exclusion criteria may be a strong point for this study.

In conclusion, we can say that* H. pylori* is not associated with iron deficiency anemia in men with normal gastrointestinal tract endoscopy results. However,* H. pylori* may be associated with iron deficiency anemia in patients with impaired gastrointestinal mucosa.

## Figures and Tables

**Table 1:** Characteristics of the study and control groups.

	Study group (*N* = 69)	Control group (*N* = 48)	*P*
Age (years)	37.4 ± 10.4	40.2 ± 10.0	0.143
*H. pylori N* (%)	45 (65.2%)	31 (64.6%)	0.896
RBC (4.2–5.1 × 10^6^/uL)	3.84 ± 0.36	4.69 ± 0.38	<0.001
Hemoglobin (12–15 gr/dL)	9.6 ± 1.3	13.4 ± 0.97	<0.001
HTC (%)	31.9 ± 3.3	40.5 ± 2.7	<0.001
MCV (80–96 fl)	71.5 ± 5.8	85.9 ± 6.2	<0.001
Iron (37–145 *μ*g/dL)	26.1 ± 12	74 ± 27	<0.001
Ferritin (13–150 ng/mL)	5.4 ± 2.8	66.7 ± 51.9	<0.001

**Table 2:** Measurements of patients in study group according to the *Helicobacter pylori* infection.

	*H. pylori* (+) (*N* = 45)	*H. pylori* (−) (*N* = 24)	*P*
RBC (4.2–5.1 × 10^6^/uL)	3.89 ± 0.37	4.00 ± 0.35	0.234
Hemoglobin (12–15 gr/dL)	9.5 ± 1.4	9.8 ± 1.2	0.466
HTC (%)	31.5 ± 3.4	32.5 ± 3.0	0.261
MCV (80–96 fl)	71.2 ± 6.1	72.1 ± 5.2	0.546
Iron (37–145 *μ*g/dL)	26.6 ± 13.7	25.0 ± 8.2	0.584
Ferritin (13–150 ng/mL)	5.5 ± 2.5	5.2 ± 3.2	0.439

**Table 3:** Measurements of patients in control group according to the *Helicobacter pylori* infection.

	*H. pylori* (+) *N* = 31	*H. pylori* (−) *N* = 17	*P*
RBC (4.2–5.1 × 10^6^/uL)	4.7 ± 0.35	4.59 ± 0.44	0.351
Hemoglobin (12–15 gr/dL)	13.5 ± 0.97	13.3 ± 0.97	0.402
HTC (%)	40.7 ± 2.6	40.2 ± 2.9	0.535
MCV (80–96 fl)	86.0 ± 6.6	85.9 ± 5.7	0.959
Iron (37–145 *μ*g/dL)	73.9 ± 30.5	75.7 ± 22.5	0.830
Ferritin (13–150 ng/mL)	59.7 ± 43.7	79.4 ± 63.8	0.213

**Table 4:** Measurements of patients according to the *Helicobacter pylori* infection.

	*H. pylori* (+) *N* = 76	*H. pylori* (−) *N* = 41	*P*
RBC (4.2–5.1 × 10^6^/uL)	4.2 ± 0.54	4.2 ± 0.46	0.803
Hemoglobin (12–15 gr/dL)	11.1 ± 2.3	11.2 ± 2.0	0.863
HTC (%)	35.3 ± 5.5	35.7 ± 4.8	0.695
MCV (80–96 fL)	77.2 ± 9.6	77.8 ± 8.7	0.747
Iron (37–145 *μ*g/dL)	45.9 ± 32.1	46.0 ± 29.7	0.788
Ferritin (13–150 ng/mL)	27.6 ± 38.5	36.0 ± 54.8	0.949
